# Validity and reliability of the Brazilian version of the Patient Dignity Inventory (PDI – Br)*

**DOI:** 10.1590/1518-8345.4015.3371

**Published:** 2021-01-08

**Authors:** Suzana Cristina Teixeira Donato, Toshio Chiba, Ricardo Tavares de Carvalho, Marina de Góes Salvetti

**Affiliations:** 1 Universidade de São Paulo, Escola de Enfermagem, São Paulo, SP, Brazil.; 2 Universidade de São Paulo, Faculdade de Medicina, São Paulo, SP, Brazil.

**Keywords:** Neoplasms, Palliative Care, Validation Study, Psychometrics, Translating, Surveys and Questionnaires, Neoplasias, Cuidados Paliativos, Estudo de Validação, Psicometria, Tradução, Inquéritos e Questionários, Neoplasias, Cuidados Paliativos, Estudio de Validación, Psicometría, Traducción, Encuestas y Cuestionarios

## Abstract

**Objective:**

to perform the psychometric validation of the Brazilian version of the Patient Dignity Inventory (PDI – Br) in patients with advanced diseases in palliative care.

**Method:**

a methodological study to verify the psychometric properties of the Patient Dignity Inventory (PDI – Br) instrument, through validity and reliability tests.

**Results:**

the exploratory factor analysis showed a factorial solution with three factors, responsible for 40.9% of the explained variance, with adequate internal consistency for the Presence of Symptoms (α=0.859), Dependence (α=0.871), and Existential Suffering (α=0.759) domains. The test-retest was performed and indicated moderate to strong correlations. Convergent validity demonstrated a positive correlation between the Presence of Symptoms and the sadness (r=0.443) and anxiety (r=0.464) variables. Weak negative correlations were observed between the PDI – Br domains and functionality, spiritual well-being and quality of life.

**Conclusion:**

composed of three domains and 25 items, the PDI – Br instrument presented satisfactory psychometric properties for its use in our environment, through the evidence of validity and reliability.

## Abstract (pt)

**Objetivo:**

realizar a validação psicométrica da versão brasileira do *Patient Dignity Inventory* (PDI – Br), em pacientes com doenças avançadas em cuidados paliativos.

**Método:**

estudo metodológico de verificação das propriedades psicométricas do instrumento *Patient Dignity Inventory* (PDI – Br), por meio de testes de validade e confiabilidade.

**Resultados:**

a análise fatorial exploratória mostrou solução fatorial com 3 fatores, responsáveis por 40,9% da variância explicada, com consistência interna adequada para os domínios Presença de Sintomas (α=0,859), Dependência (α=0,871) e Sofrimento Existencial (α=0,759). O teste-reteste foi realizado e indicou correlações moderadas a fortes. A validade convergente demonstrou correlação positiva entre a Presença de Sintomas e as variáveis de tristeza (r=0,443) e ansiedade (r=0,464). Foram observadas correlações negativas fracas entre os domínios do PDI-Br e a funcionalidade, o bem-estar espiritual e a qualidade de vida.

**Conclusão:**

o PDI-Br composto por três domínios e 25 itens apresentou propriedades psicométricas satisfatórias para o uso do instrumento em nosso meio, por meio das evidências de validade e confiabilidade.

## Abstract (es)

**Objetivo:**

realizar la validación psicométrica de la versión brasileña del *Patient Dignity Inventory* (PDI – Br) en pacientes con enfermedades avanzadas que reciben cuidados paliativos.

**Método:**

estudio metodológico de verificación de las propiedades psicométricas del instrumento *Patient Dignity Inventory* (PDI – Br), a través de tests de validez y confiabilidad.

**Resultados:**

el análisis factorial exploratorio mostró una solución factorial con 3 factores, que explican una varianza del 40,9%, con una consistencia interna adecuada para los dominios de Presencia de Síntomas (α=0,859), Dependencia (α=0,871) y Sufrimiento Existencial (α=0,759) Se realizó un test-retest que indicó correlaciones moderadas a fuertes. La validez convergente demostró una correlación positiva entre la Presencia de Síntomas y las variables de tristeza (r=0,443) y ansiedad (r=0,464). Se observaron correlaciones negativas débiles entre los dominios del PDI – Br y la funcionalidad, el bienestar espiritual y la calidad de vida.

**Conclusión:**

compuesto por tres dominios y 25 ítems, el PDI – Br presentó propiedades psicométricas satisfactorias a través de los resultados de validez y confiabilidad, que hacen adecuado el uso de este instrumento en nuestro medio.

## Introduction

The progressive aging of the population shows that people are living longer, but with a high prevalence of chronic diseases, neoplasms or dementia^(^ 1 ^-^ 2 ^)^; consequently, their quality of life decreases. According to the Brazilian Institute of Geography and Statistics (*Instituto Brasileiro de Geografia e Estatística*, IBGE, in Portuguese), in 2050 the population over 60 will be 2 billion people and 25% of them will be over 65^(^ 3 ^)^. Neoplasms also represent a public health problem and Brazil is expected to have 625,000 new cases *per* year from 2020 to 2022^(^ 4 ^)^.

However, the increase in life expectancy has not been accompanied by a better quality of life in the phenomenon of aging and illness. Technological advances and the variety of therapies available lead to a constant search for the cure of diseases, leaving interventions that focus on the dignified end of life in a second place^(^ 5 ^)^.

Differentiating itself from curative medicine, the palliative care approach is a type of assistance that proposes multifaceted care, with the management of physical, social, emotional, and spiritual symptoms for patients facing advanced life-threatening diseases^(^ 5 ^)^.

In the context of palliative care, patients can experience situations that affect their perception or sense of dignity, which can be defined as a value, from which the person is perceived by the world and by himself/herself as a valuable and respectful human being who keeps its essence intact, even when facing the physical degradation caused by different circumstances^(^ 6 ^)^.

The literature shows that the sensation of loss of dignity has been associated with an absence of the will to live, indicating a strong connection with depression, lack of hope, and expression of an interest in anticipating death^(^ 7 ^)^. Thus, the investigation of the sense or perception of dignity in the scope of palliative care has been gaining prominence in the health area.

A Brazilian study investigated the concept of dignity of patients in palliative care and showed that both health professionals and caregivers can influence self-perception of dignity. Being a “correct” person, maintaining autonomy and being cared for with respect were the elements that positively influenced the perception of dignity; on the other hand, urban violence and the lack of public accessibility policies had negative influences^(^ 8 ^)^.

Physician Harvey Max Chochinov from Canada is one of the main researchers in this area and proposed the Dignity Model, with the purpose of establishing the association between dignity and psychosocial factors in patients in an advanced stage of an incurable disease^(^ 9 ^-^ 10 ^)^. For Chochinov, maintaining dignity allows patients in palliative care to continue performing their usual roles^(^ 9 ^-^ 10 ^)^.

Based on the Theoretical Dignity Model, Chochinov proposed an instrument composed of 25 items distributed in five domains, called the Patient Dignity Inventory (PDI), in order to identify the problems associated with the loss of dignity^(^ 9 ^-^ 10 ^)^.

The Dignity Inventory proposed by Chochinov was translated, adapted and validated in Germany, Spain, Italy, Portugal and Greece^(^ 11 ^-^ 15 ^)^. So far, in Brazil there is still no single questionnaire that identifies the problems related to dignity for patients in palliative care, but there is a growing demand for this type of care.

Investigating the concept of dignity in patients in palliative care can contribute to direct the focus of care, in addition to allowing for the evaluation of interventions with the potential to improve the sense of dignity of these patients^(^ 2 ^)^.

Thus, the aim of this study was to perform the psychometric validation of the Brazilian version of the Patient Dignity Inventory (PDI – Br) in patients with advanced diseases in palliative care.

## Method

A methodological study to verify the psychometric properties of the Patient Dignity Inventory (PDI – Br) instrument, based on validity and reliability tests of the measurement instrument.

The study was set up in outpatient clinics in Palliative Care at the Cancer Institute of the State of São Paulo (*Instituto do Câncer do Estado de São Paulo*, ICESP, in Portuguese) and at the Clinical Hospital of the Medical School of the University of São Paulo (*Hospital das Clínicas da Faculdade de Medicina da Universidade de São Paulo*, HC-FMUSP, in Portuguese).

The data collection process was approved by the Ethics Committees of the Nursing School at the University of São Paulo and of co-participating institutions, under opinion No. 2,490,660. In addition, the study was carried out with the assistance of the Coordination for the Improvement of Higher Level Personnel (*Coordenação de Aperfeiçoamento de Pessoal de Nível Superior*, CAPES, in Portuguese) - Brazil, Code 001. The psychometric validation process of the Patient Dignity Inventory was authorized by Canadian psychiatrist Harvey Max Chochinov, author of the original instrument.

For selecting the sample, the non-probabilistic sampling technique was chosen for convenience. Men and women in advanced stages of incurable diseases and in palliative care, assisted in the outpatient clinics selected for the study, were invited to participate. Those who met the criteria of a minimum age of 18 years old, maintained cognitive ability (patients without evidence of delirium or dementia) and preserved verbal communication were included in the study.

The data collection process was carried out from April to June 2018. The active search for patients occurred between consultations with the palliative care team. The patients who accepted the approach signed the Free and Informed Consent Form and answered a questionnaire to identify the sociodemographic and clinical situation.

Scales were also applied to assess symptoms and quality of life. The Brazilian version of the Hospital Anxiety and Depression Scale (HADS) was used to assess anxiety and depression. The HADS scale has 14 items divided into two subscales, with scores varying from 0 to 21; scores between 0 and 10 in each subscale indicate absence of symptoms or mild changes and scores between 11 and 21 indicate moderate to severe changes^(^ 16 ^)^. The Brazilian version of the Edmonton Symptom Assessment System (ESAS - Br) was used to assess the intensity of the symptoms: pain, fatigue, nausea, depression, anxiety, drowsiness, appetite, well-being, dyspnea and sleep. Each symptom was assessed from 0 to 10^(^ 17 ^)^.

Two items from the Brazilian version of the European Organization for Research and Treatment of Cancer Quality of Life Questionnaire Core 30 (EORTC-QLQ-C30) scale were used to assess overall self-perception of health and quality of life. The scores of these items vary from 1 to 7, with 1 being very poor health and quality of life and 7, excellent^(^ 18 ^)^.

The Brazilian version of the Functional Assessment of Chronic Illness Therapy-Spiritual Well-Being (FACIT-sp 12) scale was used to assess spiritual well-being by means of two subscales: “meaning/peace” and “faith”, in which the higher score, the higher the patients› spiritual well-being^(^ 19 ^)^.

The Karnofsky Performance Scale (KPS) was used to assess the functional capacity of the patients, with a score of 100 to 0, with 100 being preserved functional capacity and 0 representing a patient in the process of death^(^ 20 ^)^, in addition to the translated and adapted version of the Patient Dignity Inventory (PDI – Br).

Construct validity was performed by exploratory factor analysis, which verifies correlations between several variables, grouping them into a set of common latent dimensions, the factors, domains or dimensions^(^ 21 ^-^ 24 ^)^. For the adequacy of the data in the factor analysis, the Kaiser-Meyer-Olkin (KMO) coefficient was used, for which the literature indicates that values above 0.80 are acceptable^(^ 24 ^)^.

The exploratory factor analysis was performed with the Statistical Package for Social Science (SPSS) statistical software, version 20.0 for Windows, through the latent structure of the relations, by the main components. The factor extraction technique was the latent root, for which eigenvalues greater than 1 are significant. The method of oblique rotation (oblimin) of the correlation matrix was used to achieve theoretically significant factors or constructs^(^ 24 ^)^.

By means of concurrent validity, the accuracy of an instrument is verified, contrasting it with a gold standard or an external criterion. This type of verification is divided into two types: convergent (when there is a correlation with the criterion) and divergent (when there is no correlation with the criterion)^(^ 25 ^)^. Concurrent validity was analyzed using Pearson’s correlation coefficient, a numerical measure that verifies the relationship between two variables. It ranges from 0 to 1, either positive or negative and the closer to 1, the stronger the correlation^(^ 24 ^)^.

In Brazil, until now, there is no model of instrument that quantifies self-perception of dignity. Based on the PDI validation carried out in Germany, Italy, Canada, and Spain^(^ 11 ^-^ 14 ^)^ and according to the literature recommendation, other scales were applied to the patients in association with the Patient Dignity Inventory (PDI), for the analysis of the correspondence of the phenomenon of dignity with external criteria.

For the test of association between dignity and physical symptoms, the Edmonton Symptom Assessment System (ESAS-Br)^(^ 17 ^)^ and the Hospital Anxiety and Depression Scale (HADS)^(^ 16 ^)^ were used. The established assumption was that the loss of dignity has a positive correlation with depression, anxiety, and physical symptoms.

The assumption was also made that the loss of dignity is negatively correlated with functional capacity, spirituality, and quality of life. In order to assess the association between loss of dignity and decreased functional capacity, the Karnofsky Performance Scale^(^ 20 ^)^, the FACIT-sp 12 scale^(^ 19 ^)^ and the two items on quality of life of the QLQ-C30 were used^(^ 18 ^)^.

The internal consistency tools were used to assess reliability, using Cronbach’s α and test-retest. Internal consistency estimates the isonomy of the items, indicating whether the items on the scale measure the same characteristic and, when they do, they are inclined to demonstrate a good correlation with each other^(^ 24 ^)^. The analysis of the *α* value also contributes to the conformation of the items, to the resolution of sustaining their permanence or the removal^(^ 14 ^)^.

The conjecture of reliability by the test-retest is based on establishing the association of the scores achieved on the same instrument by the same people at two different times and the expectation is that the scores achieved show an association^(^ 23 ^-^ 24 ^)^. In the study, the PDI – Br was used for patients in the consultation following the first approach.

## Results

In total, 135 patients were analyzed, followed-up at the ICESP (61.5%) and at the HC-FMUSP (38.5%) and were approached on average in the 4^th^ consultation; their mean age was 65 years old, with a mean schooling of 5.9 years, and most of them were retired (68.2%).

Neoplasms were predominant, accounting for 68.2% of the presentations, followed by diseases of the respiratory system (11.8%), cardiovascular diseases (6.7%), and neurological diseases (4.4%), as shown in Table 1.

**Table 1:** Identification of the patients' sociodemographic and clinical characteristics. São Paulo, SP, Brazil, 2018

Qualitative variables (n = 135)			
	n (%)		n (%)
Gender		Marital status	
Male	74 (54.8)	Has a partner	71 (52.6)
Female	61 (45.2)	No partner	64 (47.4)
Religion		Work	
Catholic	76 (56.3)	Retired	92 (68.2)
Evangelical	43 (31.9)	Illness benefit	18 (13.3)
Others	6 (4.4)	Unemployed	13 (9.6)
No religion	3 (2.2)	Pensioner	11 (8.2)
No information	7 (5.2)	No information	1 (0.7)
Diagnosis			
Neoplasms	92 (68.2)		
Respiratory system diseases	16 (11.8)		
Cardiovascular system diseases	9 (6.7)		
Neurological diseases	6 (4.4)		
Chronic renal failure	3 (2.2)		
Others*	9 (6.7)		
**Quantitative variables (n = 135)**		**Mean (SP)**	**Median**
Functionality (Karnofsky)		66.4 (16.6)	60
Age (years old)		65.0 (16.9)	66
Schooling (years)		5.96 (4.7)	5
Income (MW)^†^		1.53 (1.2)	1
Time of diagnosis (months)		75.4 (82.7)	39.5

* Skin (1.5%), hematological (1.5%), liver (1.5%), and rheumatic (0.7%) diseases, and congenital malformation (1.5%);

† MW = Minimum wage in Brazil, in 2018 = R$ 954.00

The Kaiser-Meyer-Olkin (KMO) coefficient was 0.84, showing an adequate conformation of the sample to perform factor analysis. Only items 11, 16 and 17 had lower factor loads than those indicated in the literature.

The results of the exploratory factor analysis were organized in a solution of 3 factors, or domains, for the PDI – Br. The eigenvalues found for the domains were 3.70 (A1), 3.33 (A2), and 3.19 (A3). The three factors together are responsible for explaining 40.9% of the total variance (Table 2).

**Table 2:** Correlation matrix between the items and factors/domains through factor analysis. São Paulo, SP, Brazil, 2018

Items	Domains		
	PA1	PA2	PA3
PDI 8	0.782	0.043	-0.104
PDI 7	0.773	0.048	0.009
PDI 6	0.653	0.051	0.031
PDI 5	0.478	0.221	0.118
PDI 24	0.472	-0.069	0.374
PDI 4	0.395	0.214	0.069
PDI 3	0.388	0.326	-0.113
PDI 19	0.362	0.031	0.311
PDI 9	0.356	-0.017	0.273
PDI 11	**0.284**	0.231	0.200
PDI 16	**0.277**	0.167	0.177
PDI 2	-0.028	0.922	-0.008
PDI 1	-0.003	0.874	-0.017
PDI 10	0.112	0.636	0.118
PDI 20	0.175	0.539	0.175
PDI 18	0.213	0.512	-0.008
PDI 25	-0.136	0.119	0.788
PDI 14	0.283	-0.173	0.610
PDI 23	0.123	-0.022	0.588
PDI 21	-0.087	0.122	0.554
PDI 12	-0.026	0.153	0.537
PDI 22	-0.119	0.032	0.372
PDI 13	0.086	-0.160	0.336
PDI 15	0.245	-0.069	0.307
PDI 17	0.168	0.012	**0.260**

To validate the composition of the PDI – Br in three factors/domains, correlations between the domains were performed, in pairs. The correlations between the A1 and A2 domains (r=0.433) and between A1 and A3 (0.430) were classified as moderate. The correlation between the A2 and A3 domains was weak (0.285).

To obtain the reliability of the PDI – Br, a test-retest was performed with 32 patients approached at two different times, with a mean time span of 31.6 days (SD 11.3). In addition, in the analysis of internal consistency the correlations between items in the three domains detected in the exploratory factor analysis were identified for the reliability of the instrument, described in Table 3.

**Table 3:** Internal consistency analysis (Cronbach's α) of the Patient Dignity Inventory (PDI - Br). São Paulo, SP, Brazil, 2018

Domain/α*	Items	Correlation with the other items	α value of the domain if the item is excluded
A1 (α=0.859)	3	0.442	0.854
	4	0.538	0.848
	5	0.633	0.840
	6	0.651	0.839
	7	0.705	0.834
	8	0.631	0.840
	9	0.449	0.854
	11	0.496	0.851
	16	0.439	0.855
	19	0.477	0.852
	24	0.572	0.845
			
A2 (α=0.871)	1	0.754	0.829
	2	0.789	0.820
	10	0.708	0.841
	18	0.572	**0.874**
	20	0.666	0.851
			
A3 (α=0.759)	12	0.508	0.726
	13	0.317	0.754
	14	0.613	0.705
	15	0.398	0.743
	17	0.313	**0.762**
	21	0.424	0.740
	22	0.289	0.758
	23	0.507	0.725
	25	0.619	0.709

* Cronbach's α

All the items in the A1 domain presented moderate to strong correlations, as well as the items in the A2 domain. The increase provided in α by the removal of item 18 from the A2 domain was 0.871 to 0.874. Removing item 17 from the A3 domain increased α from 0.759 to 0.762 (Table 3).

The correlation analysis of the retest for items 2, 5, 13, 14, 15, 16, 17, and 18 was strong and significant. Items 1, 3, 4, 6, 7, 8, 9, 10, 11, 19, 20, 23, 24, and 25 showed moderate and significant correlations with the answers of the first interview.

Regarding the first interview, item 22 showed perfect linear correlation (r=1, p<0.001), while item 12 showed a weak correlation with the answers of the first interview, as shown in Table 4.

**Table 4:** Correlation of the scores obtained in the PDI - Br test-retest. São Paulo, SP, Brazil, 2018

Patient Dignity Inventory, PDI - Br (n=32)			
	Items	r*	p†
1.	Not being able to perform daily life activities (e.g.: taking a bath, getting dressed).	0.557	< 0.001
2.	Not being able to perform my body functions independently (e.g.: needing help to go to the toilet).	0.755*	< 0.001
3.	Feeling stressing physical symptoms (e.g.: pain, shortness of breath, nausea).	0.508	0.003
4.	Feeling that, for the others, my appearance changed substantially.	0.602	< 0.001
5.	Feeling depressed.	0.784	< 0.001
6.	Feeling anxious.	0.643	< 0.001
7.	Feeling uncertainty about my disease and the treatment.	0.611	< 0.001
8.	Worrying about my future.	0.571	< 0.001
9.	Not being able to think clearly.	0.627	< 0.001
10.	Not being able to go on with my routine activities.	0.551	< 0.001
11.	Feeling that I am no longer who I was.	0.663	< 0.001
12.	Not feeling useful or valued.	0.330	0.065
13.	Not being able to fulfill important roles (e.g.: wife/husband, father/mother).	0.803	< 0.001
14.	Feeling that life makes no sense or has no purpose any longer.	0.703	< 0.001
15.	Feeling that I have not made any significant and lasting contribution in my life.	0.771	< 0.001
16.	Feeling that I have "unfinished businesses" (e.g.: incomplete or unsaid things).	0.718	< 0.001
17.	Worrying about my spiritual life not being significant.	0.823	< 0.001
18.	Feeling that I am a burden for the others.	0.709	< 0.001
19.	Feeling that I have no control over my own life.	0.692	< 0.001
20.	Feeling that my disease and care needs reduced my privacy.	0.598	< 0.001
21.	Not feeling supported by my group of friends and family.	-	-
22.	Not feeling support from the health professionals who care for me.	1	< 0.001
23.	Feeling that I cannot mentally "fight" against the challenges of the disease any longer.	0.633	< 0.001
14.	Not being able to accept things as they are.	0.694	< 0.001
15.	Not being treated with respect or understanding.	0.641	< 0.001

* Pearson's correlation coefficient;

† Significance level, p<0.001

The exploratory factor analysis of the PDI – Br showed that all the items were loaded in the data correlation matrix. For the decision to remove or keep the items in the final instrument, the factorial loads were evaluated together with observation of the internal consistency, using the value of Cronbach’s α*;* although items 11, 16 and 17 presented factorial loads below 0.30 (FL=0.284, FL=0.277 and FL=0.260, respectively), they remained in the instrument. Only the removal of item 17 would increase the value of Cronbach’s α by 0.003, an increase considered insignificant. The analysis of the internal consistency in association with the factorial loads is the basis for the composition of the PDI – Br in 25 items, gathered in 3 domains/factors/dimensions (A1, A2, and A3).

In the A1 domain, there are items related to physical and emotional symptoms, so it was named “Presence of Symptoms” (PS), as well as in the Canadian instrument. The A2 domain received the nomenclature of “Dependence” (D), since its conformation is identical to the Chochinov instrument. The A3 domain was named “Existential Suffering”, as it contemplates 3 of the items in the Canadian instrument domain, items 12, 13, and 14.

Moderate, positive, and significant correlations were noticed between the PS domain and the symptoms of sadness and anxiety, measured by the ESAS and anxiety, as measured by the HADS, in the observation of convergent validity. Among the other symptoms measured by the ESAS and HADS scales and the PDI – Br, the correlations were weak or very weak (Table 5).

**Table 5:** Correlation of the Patient Dignity Inventory (PDI - Br) and the ESAS, HADS, Karnofsky, FACIT- sp, and EORTC-QLQ-C30 scales. São Paulo, SP, Brazil, 2018

Scales (n = 135)	Domains (PDI - Br)					
	PS*		D^†^		SS^‡^	
ESAS	r^§^	p^||^	r^§^	p^||^	r^§^	p^||^
Pain	0.230	< 0.001	0.280	< 0.001	0.068	0.291
Tiredness	0.179	0.005	0.224	< 0.001	0.132	0.038
Nausea	0.154	0.021	0.109	0.102	0.142	0.033
Sadness	0.443	< 0.001	0.325	< 0.001	0.253	< 0.001
Anxiety	0.464	< 0.001	0.242	< 0.001	0.143	0.026
Sleepiness	0.142	0.029	0.087	0.181	0.136	0.036
Appetite	0.010	0.882	0.005	0.935	0.043	0.514
Well-being	0.131	0.044	0.015	0.820	0.066	0.311
Shortness of breath	0.119	0.067	0.140	0.031	0.176	0.007
Sleep	0.146	0.022	0.078	0.221	0.032	0.614
Total	0.372	< 0.001	0.262	< 0.001	0.223	< 0.001
**HADS**						
Anxiety	0.430	< 0.001	0.269	< 0.001	0.240	< 0.001
Depression	0.336	< 0.001	0.235	< 0.001	0.263	< 0.001
**Karnofsky (KPS)**						
	-0.257	< 0.001	-0.291	< 0.001	-0.238	< 0.001
**FACIT -sp 12**						
Meaning and peace	-0.296	< 0.001	-0.127	0.036	-0.361	< 0.001
Faith	-0.143	0.022	0.018	0.775	-0.316	< 0.001
Total	-0.254	< 0.001	-0.068	0.256	-0.374	< 0.001
**EORTC-QLQ-C30**						
General health	-0.202	0.001	-0.149	0.018	-0.206	0.001
General quality of life	-0.269	< 0.001	-0.180	0.004	-0.257	< 0.001

* Presence of symptoms;

† Dependence;

‡ Spiritual suffering;

§ Pearson' correlation coefficient;

|| p<0.05

For the PDI – Br Dependence (D) and Spiritual Suffering (SS) domains, weak and very weak correlations were found with the scales that measure symptoms. The evaluation of the hypothesis of negative correlation between the PDI – Br domains and the KPS, FACIT-sp 12 and EORTC-QLQ-C30 instruments showed negative and weak associations, as detailed in Table 5.

## Discussion

For psychometric validation studies, the literature suggests samplings over 50 individuals, recommending at least 100 people; these recommendations are necessary to guarantee more solid conclusions, from the mean of 5 or more observations per item^(^ 24 ^)^. 135 patients were interviewed in this study, which guaranteed a mean of 5.4 observations for each item of the PDI – Br.

Evidence from the literature also shows that values greater than 0.30 are admissible for factor loads^(^ 24 ^)^. The correlation matrix of the exploratory factor analysis resulted in a three-factor solution for the PDI – Br; in this analysis, items 11, 16, and 17 showed factor loads lower than the recommended ones. However, the 25 items of the instrument were organized only once in each of the three domains.

An Italian study that examined the factorial structure of the PDI applied exploratory factor analysis and also found three factors, with an explained variance of 64.4%^(^ 26 ^)^, a value which is higher than the one observed in this study (40.9%). In the Italian research the three factors were named as follows: existential suffering, psychological suffering, and physical suffering^(^ 26 ^)^. In the present study, the three factors were named presence of symptoms, dependence and existential suffering.

The definition for the final organization of an instrument should not be based only on one criterion^(^ 24 ^)^. According to the recommendations in the literature, the following elements were considered for the definition of maintenance or removal of items from the PDI – Br: factorial loads greater than 0.30, correlation between the PDI – Br domains, reliability through internal consistency (Cronbach’s α coefficient, recommended values greater than 0.70), and assessment of the domain’s α value after the removal of each item.

For a population of 135 patients, a Cronbach’s α value of 0.90 is greater than the recommended one. This indicates that the items really measure the dignity construct, indicating that there is an interconnection between them^(^ 24 ^)^.

The values found for Cronbach’s α in this study are similar to those evidenced in other PDI validation studies, such as in Canada (n=149 and α=0.93^(^ 11 ^)^), Germany (n=112 and α=0.96^(^ 12 ^)^), Spain (n=124 and α=0.89^(^ 13 ^)^), Italy (n=266 and α=0.96^(^ 14 ^)^) and Greece (n=120 and α=0.71 to 0.9^(^ 15 ^)^).

The values of the Cronbach’s α coefficient for the domains were α=0.859 (A1), α=0.871 (A2) and α=0.759 (A3), considered above what is acceptable in the literature (reference value = 0.70)^(^ 24 ^)^. The data corroborate for a three-factor solution with adequate internal consistency.

In the Canadian instrument, validated in 2008, items 10, 19, 23, and 24 were not loaded in any domain after performing the exploratory factor analysis, but were kept in the instrument, because they were highly related to a loss of sense of dignity, according to the Chochinov’s Theory^(^ 11 ^)^. In the PDI – Br all the items were loaded into the data correlation matrix after exploratory factor analysis.

For the test-retest analysis, 32 patients were approached at two different times, with a mean interval of 31 days between consultations. In the validation surveys of the Patient Dignity Inventory (PDI), the retest was performed at different intervals, 24 hours in Canada, 48 hours in Spain, 1 week in Greece, and 2 weeks in Italy^(^ 11 ^,^ 13 ^-^ 15 ^)^.

Evidence from the literature demonstrates that the reliability of the test-retest assessment can change due to approaches taken over very distant periods of time^(^ 22 ^,^ 24 ^)^. This factor may explain the lower correlation of this study, given the 31-day interval.

Palliative care patients have different stages of the disease and may progress in different proportions over the course of a month. Associated with this, there is a decrease in functionality and an increase in the number and intensity of symptoms, which may have interfered with different perceptions of dignity between the first and second approach of patients.

A research study dealt with the theme of dignity under the scrutiny of autonomy and the sense of control in patients in the final stages of an advanced disease, through a systematic review. The studies compiled by the review showed that aspects such as loss of functionality were directly related to the reduction of dignity, as the loss of body control and the development of daily activities represented a great impact for these patients^(^ 27 ^)^.

In this study, positive, moderate, and significant correlations were evidenced for the symptoms of sadness and anxiety measured by ESAS and the PDI – Br, in addition to a positive correlation between the HADS scale and the PDI – Br.

The data found for the correlation of the PDI with the symptoms of anxiety and depression were similar to those evidenced by other studies. The psychometric validation of the Canadian inventory showed a significant correlation between these elements, being moderate with anxiety (r=0.453, p<0.001) and weak with depression (r=0.374, p<0.001)^(^ 11 ^)^. In studies conducted in Germany (anxiety: r=0.66, depression: r=0.58, p<0.001) and Spain (ESAS: r=0.669 and HADS: r=0.788, p<0.001)^(^ 12 ^-^ 13 ^)^, the findings were also similar to those found in this research.

A validation study developed in Greece also found moderate and strong positive correlations between some domains of the PDI and the symptoms of anxiety (r=0.44 to 0.71; p<0.005), and weak to moderate correlations with the symptoms of depression (r=0.31 to 0.57; p<0.005)^(^ 15 ^)^. An Italian study found weak to moderate positive correlations (r=0.33 to 0.55; p<0.001) between the PDI and symptoms of depression^(^ 26 ^)^.

Evidence from the literature corroborates to explain that the loss of dignity is directly related to worse levels of anxiety and depression. A recent systematic review study found that, by raising patients’ sense of dignity, the anxiety and depression levels also improved significantly^(^ 28 ^)^.

For the symptoms evaluated by the ESAS scale, positive, weak and significant correlations were found between the symptoms of pain and tiredness, and very weak and few significant correlations between the PDI – Br and nausea, appetite, well-being, and shortness of breath. The findings are similar to those found in other validation studies conducted in Canada and Germany^(^ 11 ^-^ 12 ^)^ regarding the pain variable, for which the correlation was positive, weak, and significant.

The convergent validity tests also showed negative correlations between the inventory and the KPS, FACIT-sp 12 and EORTC-QLQ-C30 instruments, deferring the hypothesis established at first. The PDI – Br domains showed a significant although weak correlation with the functionality measured by the KPS. In the investigation carried out in Spain, the findings between the two instruments were the same^(^ 13 ^)^.

For the FACIT-sp 12 scale, the Meaning/Peace and Faith domains showed weak and significant negative correlations with the PDI – Br domains. Compared to other validation studies, in Canada the correlation found was negative, weak, and significant between the peace of mind domain of FACIT-sp 12 (r=-0.21, p<0.002) and the dignity inventory; in Spain, the correlation was moderate and negative (r=-0.442; p=0.008), the same as observed in Italy (r=-0.40; p<0.001)^(^ 11 ^,^ 13 ^-^ 14 ^)^.

For the items on the EORT-QLQ-C30 scale, weak and significant correlations were found between the domains of the PDI – Br and the questions of general health and general health quality. In Germany, the same instrument to measure quality of life was used and the correlation found was significantly moderate and negative (r=-0.42, p<0.001)^(^ 12 ^)^.

A research study carried out in 2015 in Spain showed that the sense of dignity was heightened by the application of the dignity therapy, which also brought significant beneficial effects on spiritual well-being (p<0.001) and on quality of life (p=0.011)^(^ 13 ^)^. A previous study conducted in Canada in 2002 highlighted a directly proportional relationship between better quality of life rates and a greater sense of dignity^(^ 29 ^)^.

This research corroborates with studies carried out in other countries regarding the connection between the phenomenon of dignity and the questions of anxiety, depression, pain, functionality, spirituality and quality of life. In addition, the psychometric validation data in this study are also similar to other studies of the same nature for PDI – Br.

Evidence shows that reduced samplings influence the value of Pearson’s correlation, forcing this coefficient to present a high magnitude to be significant, with values close to 1. Psychometric research studies of the PDI in larger samples should be encouraged, especially to perform the correlation hypothesis test, researching the strength of correlation between dignity and the variables measured by the other scales^(^ 22 ^)^.

The results obtained by construct validity through exploratory factor analysis, concurrent validity through convergent validity and reliability through the internal consistency and test-retest of the PDI – Br indicate satisfactory psychometric properties for its use as a measurement tool for problems related to loss of dignity.

This study has strengths and limitations, which can be transposed in new investigations. The great aspect highlighted as a strong point is the provision of a valid and reliable instrument to estimate the dignity of patients, especially those in palliative care. On the other hand, its limitation was the fact that the sample includes patients with low schooling and low socioeconomic status, which represents a portion of the population, but the results may be different in other population stratum.

## Conclusion

The tests performed demonstrate evidence of validity and reliability of the PDI – Br instrument, composed of three domains and 25 items, confirming its psychometric properties for its use in our country. This instrument offers the health professionals the possibility to assess the perception of dignity of patients in palliative care, contributing to the study of this phenomenon in the national context.
